# Morphine Suppresses Liver Cancer Cell Tumor Properties *In Vitro* and *In Vivo*

**DOI:** 10.3389/fonc.2021.666446

**Published:** 2021-04-22

**Authors:** Hao-Wen Zhang, Fei Wang, Ya-Qun Zhou, San-Ping Xu, Shi-Ying Yu, Zhan-Guo Zhang

**Affiliations:** ^1^ Department of Oncology, Tongji Hospital, Tongji Medical College, Huazhong University of Science and Technology, Wuhan, China; ^2^ School of Public Health, Tongji Medical College, Huazhong University of Science and Technology, Wuhan, China; ^3^ Department of Anesthesiology, Tongji Hospital, Tongji Medical College, Huazhong University of Science and Technology, Wuhan, China; ^4^ Health Management Center, Union Hospital, Tongji Medical College, Huazhong University of Science and Technology, Wuhan, China; ^5^ Hepatic Surgery Center, Tongji Hospital, Tongji Medical College, Huazhong University of Science and Technology, Wuhan, China

**Keywords:** liver cancer, tumorigenicity, morphine, opioids, metastasis

## Abstract

Morphine is an analgesic widely adopted to relieve cancer pain. A number of discrepancies, however, are presented by the published literature, with reports suggesting that opioids may either promote or inhibit the spread of cancer. It is of great significance to determine whether morphine may increase the risk of metastasis while utilized in liver cancer surgical treatment. In this study, we explore the effects of morphine on liver cancer cells *in vitro* and *in vivo*. Our results showed that morphine does not promote proliferative ability to cultured liver cancer cells. While morphine could increase the apoptosis rate of Hep3B/HepG2 cells. Furthermore, morphine could significantly inhibit the migratory and invasion ability of Hep3B/HepG2 cells. Subsequent investigations disclosed that morphine could inhibit sphere formation ability of Hep3B/HepG2 cells by using sphere formation assay. Based on nude mouse models, we demonstrated that morphine significantly reduced pulmonary tumorigenicity of Hep3B/HepG2 cells. In conclusion, our results found that morphine at clinical concentrations could suppress liver cancer cell tumor properties *in vitro* and *in vivo*, indicating the safety of morphine utilization in HCC patients’ pain management.

## Introduction

Hepatocellular carcinoma (HCC) has heterogeneous etiologic and molecular profiles and varied reaction to therapeutics. In spite of the progresses in operative and oncological therapies, HCC is still a worldwide burden and a major sort of liver tumor in China (1). In the United States, the incidence of HCC has nearly tripled in the past 30 years, and it is the fastest rising reason of cancer-relevant deaths (2). Recent epidemiologic researches have concentrated on potential positive effects of morphine, and the differences in cancer prognosis may originate from anesthetics on cancer biologic performance.

Perioperative care and anesthetic management are increasingly considered to be a treatment that may affect cancer recurrence, metastasis and patients survival (3–6). Over the years, clinical and laboratory studies have suggested that modifiable conditions during anesthesia and surgery may affect the recurrence of cancer (7). One of those factors is the opioid administration. It is acknowledged that opioids are widely utilized in cancer patients’ pain management, and interest in the opportunity that they may change the course of cancer has been induced (8). There has been recent focus on the management of opioids in cancer patients that were based on a combination of: numerous studies suggesting that restraining pain decreases operative stress and as a result, may possess a protective influence on neoplasm metastasis; researches both *in vitro* and *in vivo* examining the pathways that opioids can either promote or inhibit cancer (9); recent retrospective clinical experiments and ongoing prospective trials identifying whether regional anesthesia possesses the ability to prevent the metastasis of neoplasm while in comparison with general anesthesia (10). The role and underlying mechanisms of morphine in hepatocellular carcinoma, however, have been seldom studied both *in vitro* and *in vivo*.

The aim of this study was to determine the potential effects of morphine on the biologic behavior of liver cancer cells. Furthermore, we used xenograft model to investigate the *in vivo* effects of morphine on lung tumorigenicity of liver carcinoma.

## Materials and Methods

### Cell Lines and Cell Culture

Hepatocellular carcinoma Hep3B/HepG2 cells were purchased from the Chinese Type Culture Collection, Wuhan, China. Hep3B/HepG2 cells were routinely cultured in high glucose in Dulbecco Modified Eagle medium (DMEM) supplemented with 10% (v/v) fetal bovine serum (purchased from Si Ji Qing Co., Ltd., Hangzhou, China) and incubated in a humidified atmosphere of 5% CO2 plus 95% air at 37°C.

### Drugs and Treatment

Morphine hydrochloride came from Department of Anesthesiology, Tongji Hospital, Tongji Medical College, Huazhong University of Science & Technology, Wuhan, China. Apoptosis detection kit (KGA108 Annexin V-FITC/PI) was bought from Jiangsu KeyGEN Biotech Co., Ltd., Nanjing, China, which was stored at -20°C. Hep3B/HepG2 cell monolayers were incubated with morphine at clinical concentrations (0, 5, 10μM) (11) in the apoptosis analysis, Transwell assays and sphere formation assay for 24h. In the viability assay, liver cancer cells were incubated with morphine (0, 5, 10μM) for 24h, 48h and 72h to determine non-toxic concentrations. In the animal experiment, Hep3B/HepG2 cells were pretreated with morphine (0 or 10μM) for 24h before being injected into the tail vein of the nude mice.

### Viability assay

CCK-8 kit (purchased from Sigma-Aldrich, Shanghai, China) was used to evaluate the growth of liver cancer cells. Hep3B/HepG2 were cultured in 96-well flat bottom plates at the concentration of 5–10 × 103 cells per well in a volume of 100μl medium. As soon as attached to the flat, cells were treated with morphine (0, 5, 10μM) for 24h, 48h and 72h. Viability assay was conducted as described previously (12).

### Wound Healing Assay

Hep3B/HepG2 clones were grown to confluency. A linear wound was made by scraping a nonopening Pasteur pipette across the confluent cell layer, 24h after treatment by mitomycin C (10 μg/ml). Cells were washed twice to remove detached cells and debris. Then, size of wounds was observed and measured after 24h. The experiments were repeated independently three times.

### Transwell Migration and Invasion Assay

Cell migration was conducted using Transwell chambers (24-well insert, 8μm, Corning Costar, purchased from Wolcavi Biotech Co., Ltd., Beijing, China), and cell invasion was conducted using the same Transwell chambers with a Matrigel (BD Biosciences, San Jose, CA, USA). Hep3B/HepG2 cells pretreated with morphine (0, 5, 10μM) for 24h were resuspended in DMEM without fetal bovine serum (FBS) and placed into the uncoated membrane in the upper chamber at the concentration of 10^5^ cells of each chamber. DMEM supplemented with 10% FBS was used as an attractant in the lower chamber. After being incubated for 24h, cells migrated across the membrane were fixed with 4% paraformaldehyde and stained with a 4 g/L crystal violet solution. The images were observed by microscope (Keyence, Japan), and we counted nine random fields at 10× magnification. The experiments were repeated independently three times.

### Apoptosis Assay

Hep3B/HepG2 were dealt with the concentrations of morphine indicated. Then we stained samples from three experimental groups at different dose of morphine with FITC Annexin V and PI at 25°C in the dark for 15 min. Apoptotic cells were analyzed by a FACSCalibur Cytometer (Becton Dickinson, USA) within 1 h according to the manufacturer’s instructions. The experiments were repeated independently three times.

### Sphere Formation Assay

Ultralow attachment plates (bought from Corning Incorporated, Shanghai, China) were used to perform Sphere formation assay. Hep3B/HepG2 pretreated with morphine (0, 5, 10μM) were resuspended with medium supplemented with 20 ng/ml EGF, 20 ng/ml bFGF, and 2% B27 at the density around 1000cells/ml and cultured at 37°C in 5% CO2. After 1 week, we counted the spheres greater than 50μm diameter at 40 x magnification under Keyence microscope. We calculated the number of spheres per 1000 cells to evaluate Sphere formation efficiency (SFE). The experiments were repeated independently three times.

### *In Vivo* Xenograft Assay

We ordered the 5 week-aged female nude mice from the Chinese Academy of Medical Sciences Institute of experimental animals. Under the protocol on laboratory animals of the National Institutes of Health guidelines (NIH publication 96-01, 1996 revision) approved by the Institute Research Ethics Committee at the Tongji Medical College, Huazhong University of Science & Technology, all animal experiments were performed. We harvested the Hep3B/HepG2 cells expressing Luciferase. Then they were resuspended in PBS at the concentration of 1.6 ×10^7^ cells/ml. Each mouse was injected with 50μl of the cell suspension through the tail vein. Fluorescence imaging was tracked by Xenogen IVIS Imaging System (Caliper Life Sciences, USA) and the Luciferase activity was captured once a week. After 5 weeks the ending point was reached, we sacrificed all the mice and harvested their lungs for pathology analysis.

### Western Blot

Western blot analysis was performed as described previously (13). Primary antibodies include *OGFR* (Abcam, ab1717), μ-opioids receptor (Abcam, ab134054), *uPA* (Abcam,ab218106), *MMP-9* (Abcam, ab76003). Anti-β-actin (Abcam, ab8226) was used as an internal control. Immune complexes were visualized using the Beyo ECL Plus.

### RNA and Reverse Transcription-PCR

The cDNA was created according to the manufacturer’s protocol (Takara, PrimeScript RT Master Mix). Quantitative PCR was performed on StepOne Real-Time System (Bio-rad) using SYBR Premix Ex TaqTM (Takara, DRR081A) according to the manufacturer’s protocol. Gene expression was normalized to β-actin mRNA content for human genes, and expressed relatively to the control condition of each experiment. The relative expression of each target gene was determined from replicate samples using the 2^-ΔΔCt^ (Ct, cycle threshold).

### Statistical Analysis

GraphPad Prism 4.1 software (GraphPad Software, La Jolla, CA) was employed for all statistical analysis and output of figures. Statistical analyses were performed by Student’s t-test (two groups) or one-way analysis of variance followed by the Dunnett *post hoc* test (multiple groups). All data are expressed as mean ± SE from at least three separate experiments performed in triplicate except otherwise noted. The statistical significance level was defined as P<0.05.

## Results

### Morphine Does Not Promote Proliferative Ability to Cultured Liver Cancer Cells

**Figure 1:**
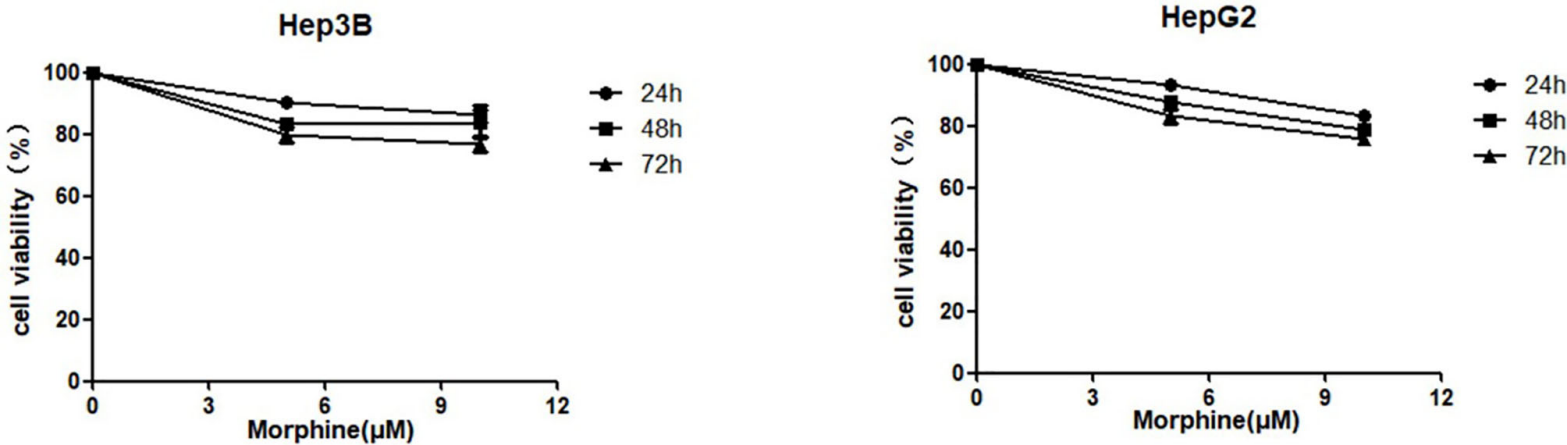
Morphine does not promote proliferative ability to cultured liver cancer cells. Hep3B/HepG2 cells were pretreated with morphine at the concentration of 0, 5, 10μM for 24h, 48h and 72h. Cell viabilities were measured by CCK-8 assay. Data are expressed as mean ± SE; n=3.

We investigated whether morphine had an effect on the viability of Hep3B/HepG2 cells. Cells were respectively pretreated with morphine (0, 5, 10μM) for 24h, 48h and 72h, followed by CCk-8 assay. Our results indicated that morphine did not promote proliferative ability to Hep3B/HepG2 cells (**Figure 1**). Meanwhile, we found that morphine has no effect on proliferative behavior of human hepatocellular carcinoma cells when applied to Hep3B/HepG2 cells under 5 or 10μM concentration for 24h. Thus, we used 24h as the checkpoint in the following experiments.

### Morphine Could Significantly Increase the Apoptosis Rate of Hep3B/HepG2 cells

**Figure 2:**
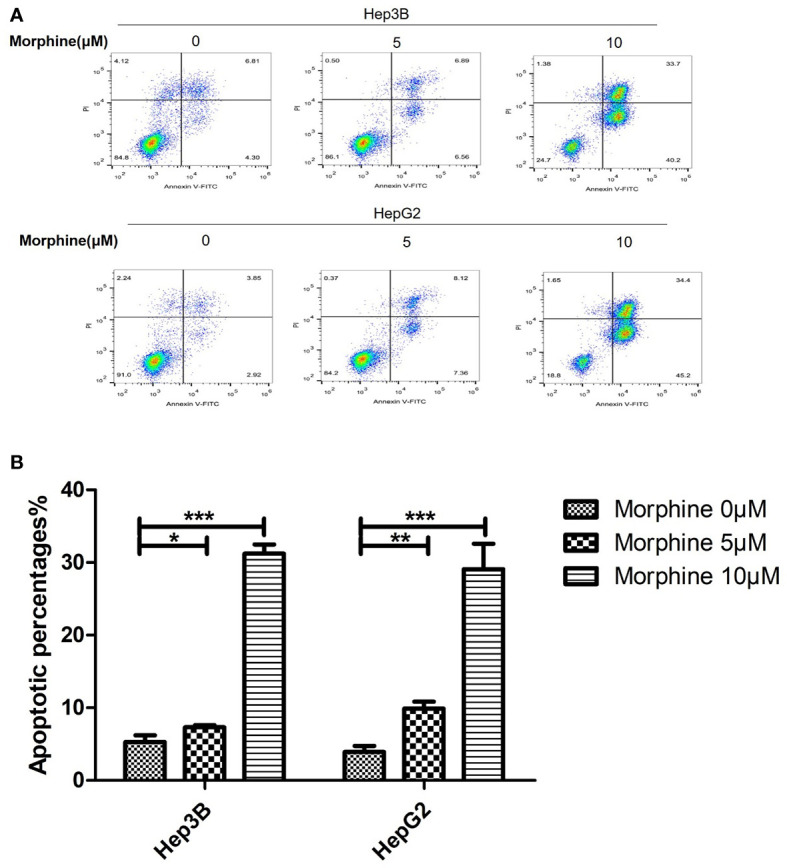
Morphine promotes the apoptosis of Hep3B/HepG2 cells *in vitro*. **(A)** Representative image of flow cytometry analysis of Annexin V and Propidium iodide as a measure of apoptosis and **(B)** quantitative analysis of apoptosis in Hep3B/HepG2 cells exposed to morphine for 24h. Data are expressed as mean ± SE; n=3. *P < 0.05, **P < 0.01, ***P < 0.001.

The apoptosis rate of Hep3B/HepG2 cells exposed to morphine had significantly increased compared with cells not exposed to morphine (**Figures 2A, B**). Those cells pretreated with 10μM morphine for 24h had the most apoptotic rate compared with other groups.

### Hep3B/HepG2 Pre-Exposed to Morphine Had Lower Migratory and Invasion Capacity *In Vitro*

**Figure 3:**
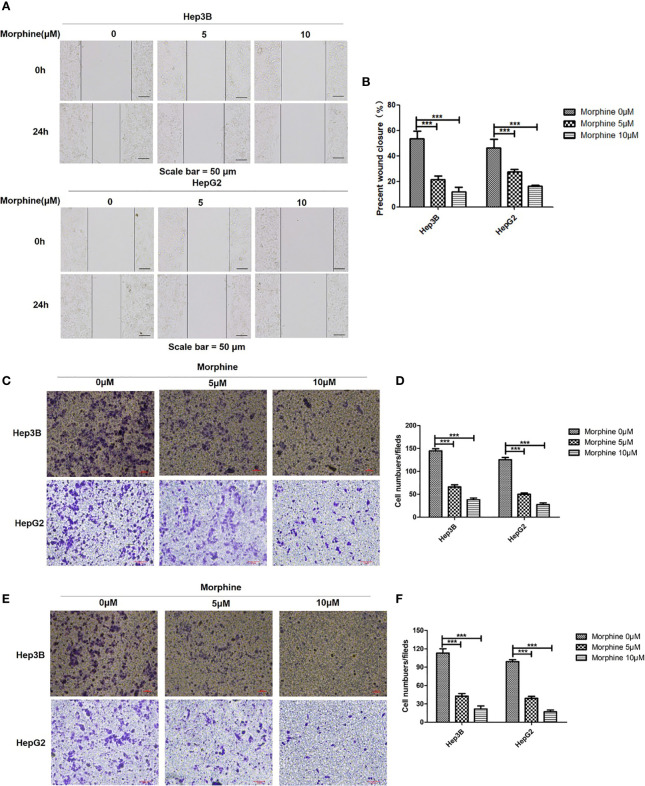
Morphine suppresses the migration and invasion ability of Hep3B/HepG2 *in vitro*. **(A)** Representative image of wound healing assay and **(B)** quantitative analysis of wound healing assay of Hep3B/HepG2 exposed to morphine for 24h. **(C)** Representative image of transwell analysis of migration and **(D)** quantitative analysis of migration of Hep3B/HepG2 exposed to morphine for 24h. **(E)** Representative image of transwell analysis of invasion and **(F)** quantitative analysis of invasion of Hep3B/HepG2 exposed to morphine for 24h. Data are expressed as mean ± SE; n=3. ***P < 0.001.

There was decreased migratory and invasion capacity of Hep3B/HepG2 pretreated with morphine compared with those without morphine. Those cells exposed to 5μM morphine or 10μM morphine for 24h had a lower number of migrating cells compared with control group (**Figures 3A–F**).

### Morphine Inhibits Sphere Formation Ability of Hep3B/HepG2

**Figure 4:**
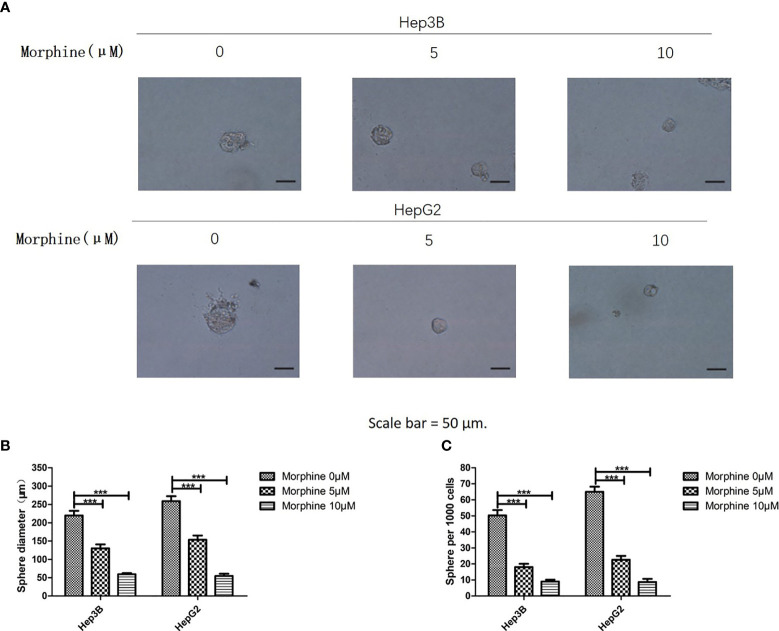
Morphine inhibits the sphere formation ability of Hep3B/HepG2 cells. **(A)** Representative pictures of spheres formed by Hep3B/HepG2 cells after treating with morphine (0, 5, 10μM) for 24h, respectively (Scale bars, 50μm). **(B, C)** Bar diagrams showed the diameter and number of spheres (spheres > 50μm). Data are expressed as mean ± SE; n=3. ***P < 0.001.

Hep3B/HepG2 cell lines were chosen to investigate the potential role of morphine in cancer sphere formation ability. We used sphere formation assay in liver cancer cells. Sphere formation assay has been applied as a representative indicator of cancer stem cell activity (14). We pretreated Hep3B/HepG2 cells with morphine (0, 5, 10μM) followed by a sphere formation assay. Results demonstrated that morphine significantly decreased the SFE and the sphere size in Hep3B/HepG2 cells (**Figures 4A–C**).

### Morphine Decreases the Tumorigenicity of Liver Cancer in Lung

**Figure 5:**
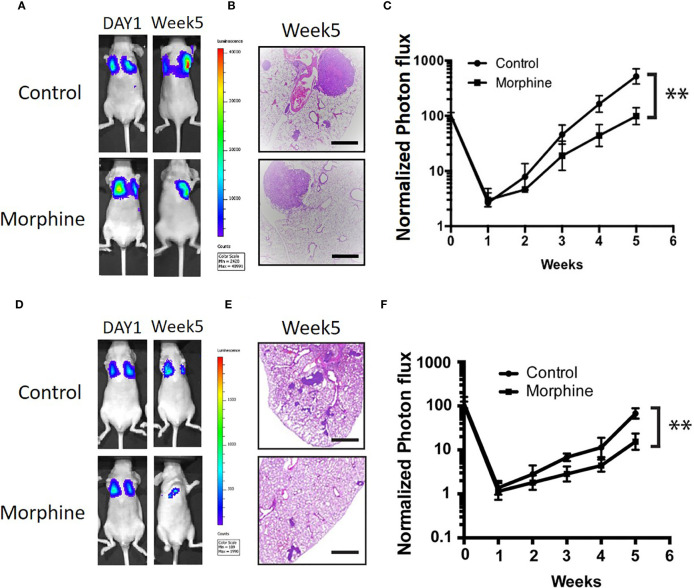
Morphine inhibits lung tumorigenicity of Hep3B/HepG2 cells *in vivo*. **(A)** Luminescence of nude mice that were tail vein injected with Hep3B cells at the initiation time point and 5 weeks after. **(B)** Lung sections of nude mice that were tail vein injected with Hep3B cells for H&E staining (scaled bar length = 500μm). **(C)** Relative quantification of luminescence of nude mice that were tail vein injected with Hep3B cells was showed. **(D)** Luminescence of nude mice that were tail vein injected with HepG2 cells at the initiation time point and 5 weeks after. **(E)** Lung sections of nude mice that were tail vein injected with HepG2 cells for H&E staining (scaled bar length = 500μm). **(F)** Relative quantification of luminescence of nude mice that were tail vein injected with HepG2 cells was showed. Data were shown as mean ± SD. n=9 per group. **P < 0.01.

In vivo experiment was performed by tail vein injection of Hep3B/HepG2 cells pretreated with or without morphine to elucidate whether morphine could decrease the tumorigenicity of liver cancer in lung. We labeled Hep3B/HepG2 with luciferase, which made it feasible for tracking and quantification of cancer cells. We found that morphine reduced the lung tumorigenicity of Hep3B/HepG2 cells significantly (**Figure 5**).

### Morphine Could Suppress Malignant Behavior of Liver Cancer Cells *via* Up-Regulation of the *OGFR* and Down-Regulation of μ-opioids Receptor (*MOR*), *uPA* and *MMP-9*

**Figure 6:**
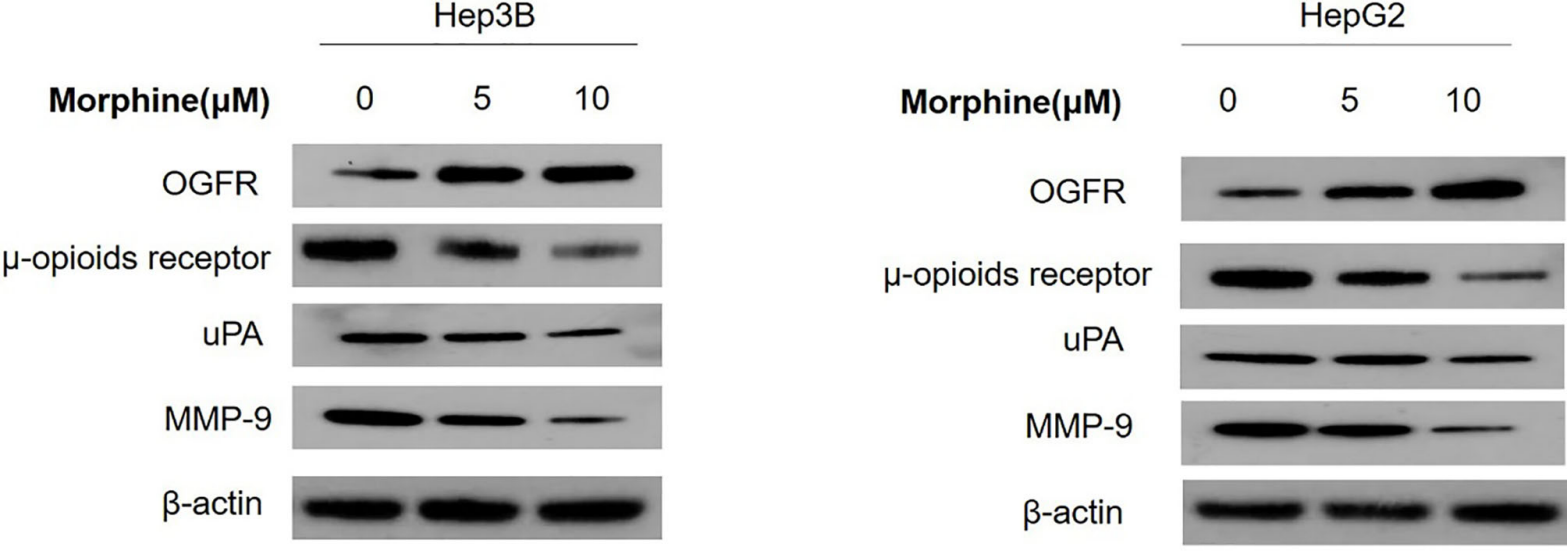
Liver cancer cells were pretreated with different concentrations of morphine (0,5,10μM) for 24h followed by Western Blot to detect the protein level of *OGFR*, *MOR*, *uPA* and *MMP-9* in Hep3B/HepG2 cells. n=3.

**Figure 7:**
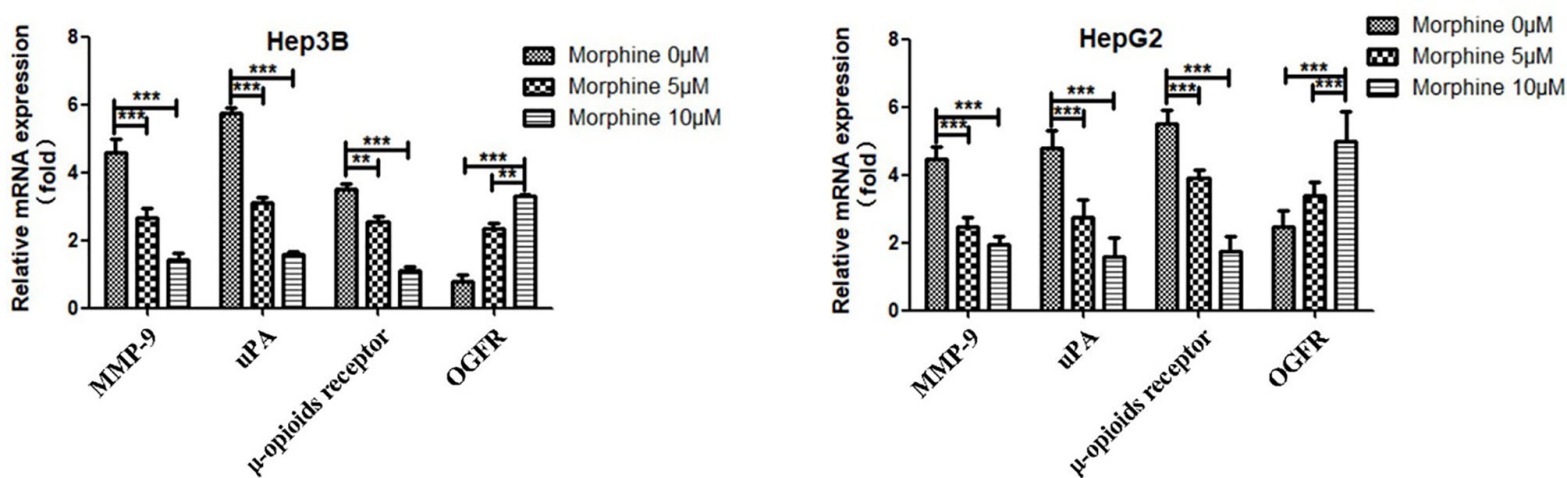
Liver cancer cells were pretreated with different concentrations of morphine (0,5,10μM) for 24h followed by qRT-PCR to detect the mRNA level of *OGFR*, *MOR*, *uPA* and *MMP-9* in Hep3B/HepG2 cells. Data are expressed as mean ± SE; n=3. **P < 0.01, ***P < 0.001.

We detected the protein and mRNA level of *OGFR*, *MOR*, *uPA* and *MMP-9* in Hep3B/HepG2 cells to explore the molecular mechanisms through which morphine could affect the liver cancer cells. Our results showed that morphine could suppress malignant behavior of liver cancer cells by up-regulation of the *OGFR* and down-regulation of *MOR*, *uPA* and *MMP-9* (**Figures 6** and **7**).

## Discussion

OPIOIDS including morphine are routinely adopted in pain management during perioperative period but have been linked to various pro- and anti-tumor properties (15). In this study, we investigated the effects of morphine on the tumor properties of liver cancer cells. We found that Hep3B/HepG2 pre-exposed to morphine does not possess higher growth potential compared with cells not exposed to morphine. Meanwhile, morphine could increase the apoptosis rate of Hep3B/HepG2 cells. Our findings further demonstrate that morphine inhibits cancer sphere formation ability due to significantly decreased sphere size and number. Furthermore, cells pre-exposed to morphine had significant lower migratory and invasion capacity *in vitro* and migrated less to the lung *in vivo*.

Former studies have also indicated that morphine-mediated influence on cancer progression may be analgesia independent (16). For instance, a number of *in vitro* studies indicate that morphine could directly affect cellular pathways controlling cell migration, survival and proliferation in breast (17–22), lung (23–25) and prostate cancer cell lines (26). So far, the majority of these studies demonstrate that morphine boosts neoplasm cell migration, survival and proliferation while just a small part of them imply a tumor-inhibiting impact of morphine *in vitro* (17, 24, 25). Our results reveal that morphine does not have a significant positive effect on proliferation of liver cancer cells *in vitro*. These contradictions may be interpreted with selection of different cancer cell lines. In consistent with our findings, Chris et al. (16) suggest that morphine does not lead to the progression of breast cancer in preclinical models for metastatic invasive lobular. The possibility that anesthetic techniques may differentially affect disease progression because of inherent drug-relevant characteristics is not excluded in our findings. Therefore, our results suggest that opioid analgesics are not inappropriate for perioperative pain management related to HCC.

General and regional anesthetics antitumor effects on various liver tumors (27) and other solid tumors have been extensively documented. However, questions have been raised regarding the possible mechanisms of opioids-induced tumor suppression. It has been reported that down-regulation of the μ-opioids receptor (*MOR*) resulted in the increase of the mitogen-activated protein kinase kinase (*MKK7*) expression and c-Jun N-terminal kinase (*JNK*) activation which, in turn, induces substrates such as pro-apoptosis proteins (28). Therefore, we propose that down-regulation of *MOR* is the key to inhibit human liver cancer progression. Our follow-up experiments revealed that this pathway may play important role in the interaction between morphine and liver cancer cells. Moreover, it has been demonstrated that opioid growth factor receptor (*OGFR*), a negative regulator of cell proliferation is involved in subsequent morphine-induced lung cancer growth inhibition. Lung cancer tissues and cell lines expressing *OGFR* which interacts with morphine may restrain lung cancer progression (29). Our results also showed up-regulation of the *OGFR* in liver cancer cells pre-exposed to morphine indicating that similar mechanism may take part in different cell lines.

Our present data reveal that Hep3B/HepG2 cells exposed to morphine had lower migratory and invasion capacity *in vitro*. We hypothesize that the effect of morphine would be mediated by the reduction level of *MMP-9* and *uPA* (30). The production of *MMP-9* and *uPA*, which are ECM-degrading enzymes, is up-regulated by neoplasm. This tendency can be reversed by morphine. *MMP-9* modulate cell proliferation, adhesion, apoptosis and differentiation and play an important part in tumor growth, angiogenesis and metastasis (31). There is an obvious link between the expression level of *uPA* receptor (*uPAR*) on the cell surface and proliferative, adhesive and migratory capacities of cancer cells. Thus, decreased level of *MMP-9* and *uPA* leads to inhibition of lung metastasis *in vivo*. Our studies showed morphine could lead to down-regulation of *MMP-9* and *uPA* indicating our assumption is reasonable.

More importantly, animal experiments indicated that morphine suppressed tumorigenicity in hepatocellular carcinoma. We chose a mouse model that employed an opioid dose similar to what Hep3B/HepG2 cells receive. The injection of liver tumor cells in the tail vein was conducted to imitate the presence of cancer cells in the circulation during and after tumor resection. Our experiments illustrate that morphine led to less lung colonization and inhibited the growth of Hep3B/HepG2 cells introduced into the blood stream of mice *via* tail vein injection. These results are in consistence with results from other experiments indicating that morphine depresses tumor progression *in vivo* (32–34). Anti-tumor effects of morphine detected in our mice model could be mediated through communication between nonmalignant cells and cancer cells by paracrine in the neoplasm microenvironment. Further studies are required to clarify the mechanisms of morphine-induced suppression of the tumorigenicity of hepatocellular carcinoma *in vivo*.

Our results demonstrate that morphine restrains cancer sphere formation ability in Hep3B/HepG2 with sphere formation assay, which contradicts with studies showing morphine promotes those in breast cancer (35). Explanations for these discrepancies may include different experimental conditions, wide ranges of morphine and differences in tumor types. Although the mechanism by which morphine drives sphere formation ability remains unclear, our study suggests that this common opioid may inhibit the sphere formation ability in hepatocellular carcinoma, potentially applying to other malignant liver tumor subtypes.

However, it is acknowledged that our study lacks an in-depth analysis of the effects of morphine to HCC. Other opioids, including fentanyl and codeine, may also mediate similar effects, and the effect of those agents on liver cancer cells needs to be analyzed in a similar fashion. Future studies should evaluate the effects on different cell lines, specifically in primary human hepatocellular carcinoma cell lines.

## Conclusions

In conclusion, our studies demonstrate that morphine does not promote the proliferative ability while increase the apoptosis rate of Hep3B/HepG2 cells. Furthermore, the present data demonstrate that exposure to morphine under relevant conditions can suppress the migratory or invasion capacity and cancer sphere formation ability *in vitro*. In the end, the animal experiment provides preliminary evidence suggesting that morphine may have potentially beneficial effects in liver neoplasm by reducing tumorigenicity efficiency.

## Data Availability Statement

The original contributions presented in the study are included in the article/supplementary material. Further inquiries can be directed to the corresponding authors.

## Ethics Statement

The animal study was reviewed and approved by Institute Research Ethics Committee at the Tongji Medical College, Huazhong University of Science & Technology.

## Author Contributions

Conception and design: S-YY and S-PX. Experiment conduction, data analysis, illustrations and manuscript writing: H-WZ, Y-QZ, and FW. Final approval of manuscript, and financial support: S-PX, S-YY, and Z-GZ. All authors contributed to the article and approved the submitted version.

## Funding

This work was supported by grants from National Natural Science Foundation of People’s Republic of China (grants 81400917, 81371250, 81502530, 81571053).

## Conflict of Interest

The authors declare that the research was conducted in the absence of any commercial or financial relationships that could be construed as a potential conflict of interest.
